# Binge-eating and sodium bicarbonate: a potent combination for gastric rupture in adults—two case reports and a review of literature

**DOI:** 10.1186/s40337-022-00677-9

**Published:** 2022-11-09

**Authors:** You Jin Han, Susmit Roy, Ashley Maria Pei Ling Siau, Adeeb Majid

**Affiliations:** 1 School of Medicine and Public Health, University of Newcastle, University Drive, Callaghan, Newcastle, NSW 2308 Australia; 2 Calvary Mater Hospital, Newcastle, Edith & Platt St, Waratah, Newcastle, NSW 2298 Australia; 3 University of Newcastle, Newcastle, Australia

**Keywords:** Stomach rupture, Gastric dilatation, Sodium bicarbonate, Feeding and eating disorders

## Abstract

**Background:**

Disordered eating behaviour including binge-eating often results in significant medical conditions, which are at times fatal. It can result in acute gastric dilatation which can lead to ischemic necrosis and stomach rupture. Dyspepsia and bloating are common symptoms following binge eating. Patients commonly use over-the-counter medications like sodium bicarbonate or home remedies for relief. However, in very rare, reported cases, sodium bicarbonate has been attributed to cause acute gastric dilatation and spontaneous gastric rupture instead.

**Methods:**

We report two cases of spontaneous gastric rupture following consumption of sodium bicarbonate containing antacids after a large meal, and a review of the literature of similar cases.

**Results:**

A total of 36 cases were identified. Approximately half of the cases (47.2%) were correlated with eating disorders, with higher prevalence in females (69%) and a very high mortality rate (41.6%). Amongst the 36 cases, sodium bicarbonate ingestion was associated with 10 cases. The lesser curvature (36.1%) and anterior wall (33.3%) are the most common sites of rupture. Associated causes include binge-eating, gas release from sodium bicarbonate, gastric content fermentation, proximal and distal outlet obstruction, and muscular atony.

**Discussion:**

Sudden distension and impaired emptying mechanism of the stomach is necessary for spontaneous gastric rupture to occur. Acute gastric dilatation with perforation requires definitive surgical management. There should be a low threshold of suspicion for patients presenting with severe abdominal pain and abdominal distension following an episode of binge-eating. There is a need for patient education around the use of over-the-counter medications or home remedies.

## Plain English summary

Disordered eating ranges from simple dieting to eating disorders. A large proportion of overweight or obese adolescents and young adults engage in disordered eating behaviours, most commonly binge-eating. While such behaviours can result in significant medical conditions, it commonly results in uncomfortable gastrointestinal symptoms. Over-the-counter medications are often used to relieve symptoms like indigestion, abdominal pain, and heartburn. Sodium bicarbonate, a common ingredient in these medications, has been associated with spontaneous stomach rupture. Despite this, sodium bicarbonate is suggested online as a home remedy for these symptoms. In this article we report two such cases of spontaneous stomach ruptures following the consumption of sodium bicarbonate containing antacids, following a large meal. We then review the literature for similar cases. We discuss the functional changes in the body resulting in the injury, the mortality rates, suggested management approaches and the relevance of our study. Our article emphasises the need for a low threshold of suspicion for patients presenting with severe abdominal pain with abdominal distension following a binge-eating episode. There is also a need to educate patients surrounding the use of over-the-counter medications or home remedies, as they are often unaware of potentially fatal adverse effects with inappropriate use.

## Introduction

Disordered eating includes a spectrum of eating-related problems, from simple dieting to eating disorders [1]. Disordered eating behaviour (DEB) can be defined as dysfunctional eating practices that do not meet the full diagnostic criteria of an eating disorder, including but not limited to practices such as bingeing and purgative practices [2]. While there is limited data about the true prevalence of DEB, studies indicate that a large proportion of overweight or obese adolescents and young adults engage in DEB [3, 4]. Amongst the different DEBs, bingeing behaviour is common. A quarter of overweight and obese children and adolescents are estimated to binge or engage in loss of control eating [5]. According to data from 2013 to 2014, approximately a third (31.6%) of Australian adolescents engage in DEB, and nearly a third engage in binge-eating (29.7%) [6]. These teenagers are also more likely to experience clinical mental health problems. Apart from mental health comorbidities, DEB is of significant medical concern as it is associated with increased risk of alcohol and tobacco use, poor nutritional intake and quality, and significant weight gain over time [4]. Amongst the myriad of medical consequence associated with DEB, an uncommon but serious and potentially fatal complication includes acute gastric dilatation (AGD) with or without gastric rupture [7]. This can occur following an episode of binge-eating or having a heavier meal after prolonged fasting [8]. The first known case of gastric rupture associated with AGD was described by Evans [7] in 1968, in a patient with anorexia nervosa. Since then, several cases of gastric rupture associated with AGD following a binge-eating episode have been described in literature [9–11].

Patients with DEB frequently complain of gastrointestinal symptoms [12] such as heartburn, bloating, and upper abdominal pain [13]. In patients with eating disorders, functional heartburn and functional dyspepsia are common, occurring in 22–51% and 23–45% respectively [14, 15]. In bulimic patients, bloating is the most common symptom reported (74.4%) [16], likely secondary to their binge-eating episodes. These gastrointestinal symptoms cause pain and discomfort to the individual, some of whom may attempt to seek instant relief with medications prescribed by their doctor. However, due to the stigma and guilt associated with DEB, particularly binge-eating [1, 2], patients may not seek medical advice and instead turn to home remedies, over-the-counter (OTC) medications, or solutions found online. A quick search online may return some of the following home remedies: trying ginger, mixing baking soda with water, or taking liquorice supplements. OTC medications targeted at treating dyspepsia and heartburn include antacids, some of which may include sodium bicarbonate. While these home remedies and OTC medications may be safe at recommended doses, patients may not fully understand the adverse effects, including risk of fatality, associated with excessive doses [17–19]. Baking soda or sodium bicarbonate is commonly available, has been associated with causing spontaneous gastric rupture [20]. Several case reports have been published, drawing the association between sodium bicarbonate ingestion, AGD, and gastric rupture [21, 22].

We present two cases of patients with an acute gastric rupture at our institution with history suggesting the consumption of sodium bicarbonate-containing antacids after an episode of binge-eating, and a review of literature for similar cases.

### Case report

#### Case 1

A 21-years-old woman was brought in by ambulance to the Emergency Department of a peripheral hospital in obvious distress, with complaints of sudden onset severe epigastric pain and vomiting following an episode of binge-eating. She had taken laxatives and sodium bicarbonate at home to relieve the abdominal discomfort. Her symptoms worsened and she was brought to the hospital urgently.

On examination, her abdomen was distended with generalised tenderness without any obvious signs of peritonism. However, the pain had not improved with opioids and methoxyflurane administered in the ambulance en-route to hospital.

Her initial observations were within normal range, but venous blood gas analysis showed acidosis with a pH of 7.284, PCO_2_ of 62.6 mmHg, and a lactate of 1.49 mmol/L. Her opioid requirements were increasing and within half an hour of presentation, she developed global board-like rigidity. Repeat pathological investigations revealed worsening acidosis. A transfer was organised to our tertiary hospital for an urgent surgical review for further management.

**Fig. 1:**
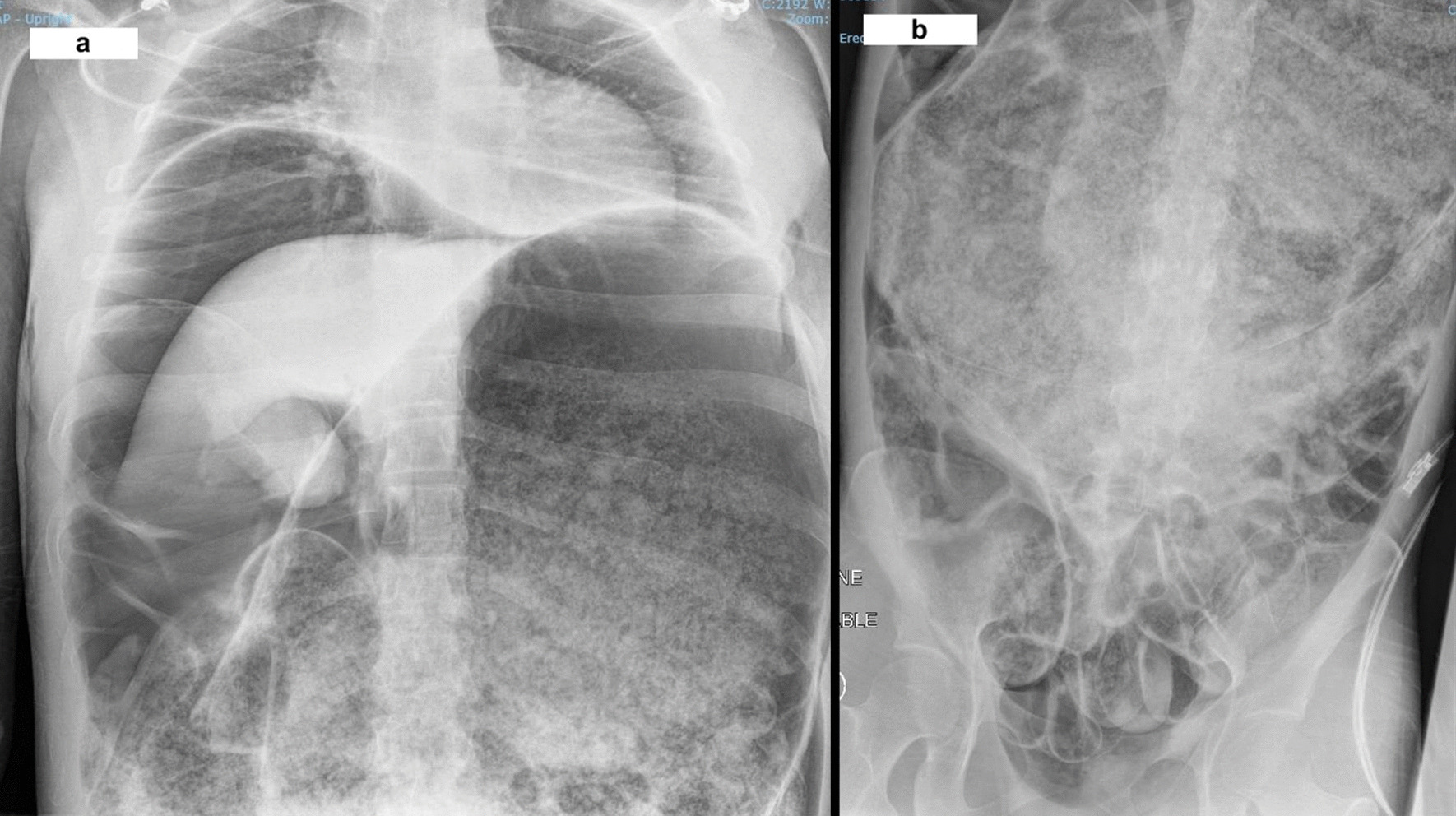
**a** Portable X-ray of chest showing pneumoperitoneum and markedly dilated stomach. **b** Portable X-ray of abdomen showing grossly dilated stomach to pelvis

Clinically, her condition deteriorated en-route and required high dosage of opioids. On arrival to the Emergency Department at our hospital, she was found to be unconscious, mottled in appearance, with a markedly distended and rigid abdomen. Further examination suggested that her legs were completely ischaemic consistent with lack of venous return secondary to inferior vena cava occlusion. At this point, her acidosis had worsened further with a pH of 6.99, PCO_2_ of 117 mmHg, and a lactate of 2.2 mmol/L. An urgent portable chest X-ray (Fig. 1A) revealed pneumoperitoneum and a markedly dilated stomach (Fig. 1B). She underwent a rapid sequence intubation with ketamine and rocuronium, and a needle decompression of her abdomen in the Emergency Department to improve cardiac output before being transferred to the operation theatre for an urgent laparotomy.

Laparotomy revealed an extremely dilated stomach filled with partially digested food material. After decompression, a large perforation was identified on the posterior/medial wall of the stomach involving the lesser curvature, measuring around 15 cm surrounded by ischaemic changes. The small and large bowels were dilated but no ischaemic changes or perforation were noted. A damage control operation was performed. Because of the nature of the perforation and non-viable stomach, a total gastrectomy and washout was performed. The ends of the oesophagus and the duodenum were stapled off and laparostomy was performed using an abdominal vacuum dressing. The entire procedure was extremely challenging as the patient was unstable and required maximum inotropic support, intravenous fluids, and blood. She was transferred to the intensive care unit postoperatively with multiple organ dysfunction and a plan for a relook laparotomy after correcting her organ dysfunction. Unfortunately, she passed away the following morning due to uncontrolled disseminated intravascular coagulopathy and multi-organ failure.

Her past medical history included a diagnosis of binge-eating disorder 3 years prior to her acute episode. She engaged in regular binge-eating followed by fasting for up to 24 h. On histological examination, no pathological lesions were found on the stomach wall except for ischaemic changes related to the rupture.

#### Case 2

A 63-years-old man self-presented to the Emergency Department of our hospital with complaints of worsening epigastric pain associated with sweating and multiple small volume vomits.

On presentation, the patient reported that he was previously well during the day and has a past medical history of myocardial infarction with percutaneous stenting and gastro-oesophageal reflux disease. He had consumed a large Mexican dinner of nachos accompanied by a full bottle of red wine. He went to bed and was awoken by epigastric pain for which he took a tablet of Esomeprazole 20 mg. However, the pain did not subside, and he ingested some baking soda (quantity he could not recollect). He mentioned that he remembered baking soda being used as a home remedy for symptoms of dyspepsia or indigestion. However, his symptoms worsened following the ingestion of baking soda.

On presentation, he was diaphoretic, and his abdomen was very tender on palpation with guarding in the upper abdomen. His observations were within normal limits. Considering his previous history of myocardial infarction, he was initially worked up with concerns of acute coronary syndrome. However, serial ECG showed no dynamic ST changes. A venous blood gas analysis revealed a normal pH of 7.377, PCO_2_ of 52.4 mmHg, but a high lactate of 3.2 mmol/L.

**Fig. 2:**
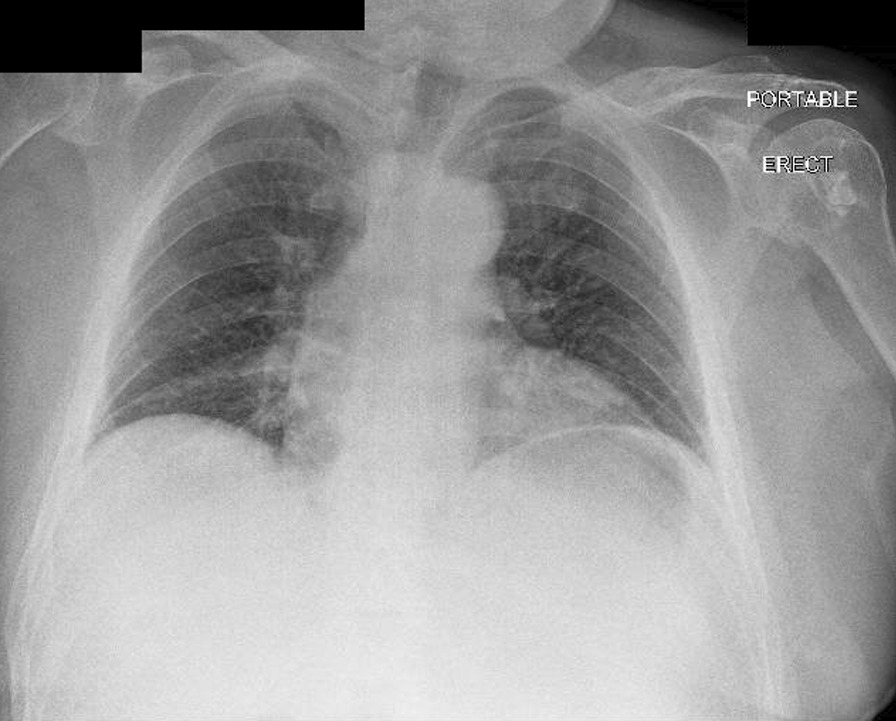
Portable X-ray of chest done in emergency department

**Fig. 3:**
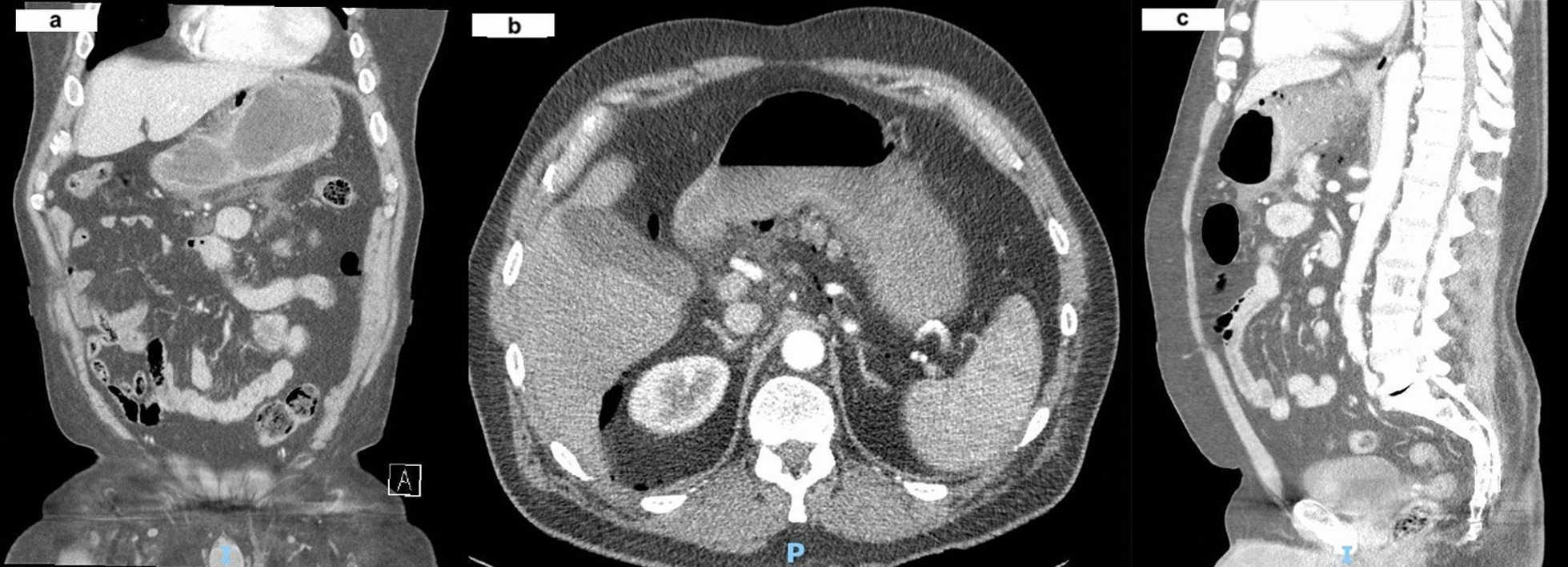
**a** Axial slice of abdomen CT showing free air in peritoneal cavity. **b** Coronal slice of abdomen CT showing free air near the lesser curvature. **c** Sagittal slice of abdomen CT showing subhepatic free air

An urgent portable chest X-ray (Fig. 2) suggested pneumoperitoneum, with air under the left hemidiaphragm. Considering the patient’s vitals were stable, a Computed Tomography (CT) scan of the abdomen and pelvis was performed (Fig. 3A–C), revealing moderate amounts of extraluminal gas in the upper abdomen, both intraperitoneally and retroperitoneally, best seen along the lesser curve of the gastric body. Small amounts of gas and fluid were noted between the pancreatic body and stomach (Fig. 3A, B), and in the subhepatic space (Fig. 3C). A surgical review initially diagnosed him with a perforated peptic ulcer. He was initiated on triple antibiotics therapy of Intravenous ampicillin, metronidazole, and gentamicin. A nasogastric tube was placed with suction to decompress the stomach, and he was scheduled for urgent operation.

**Fig. 4:**
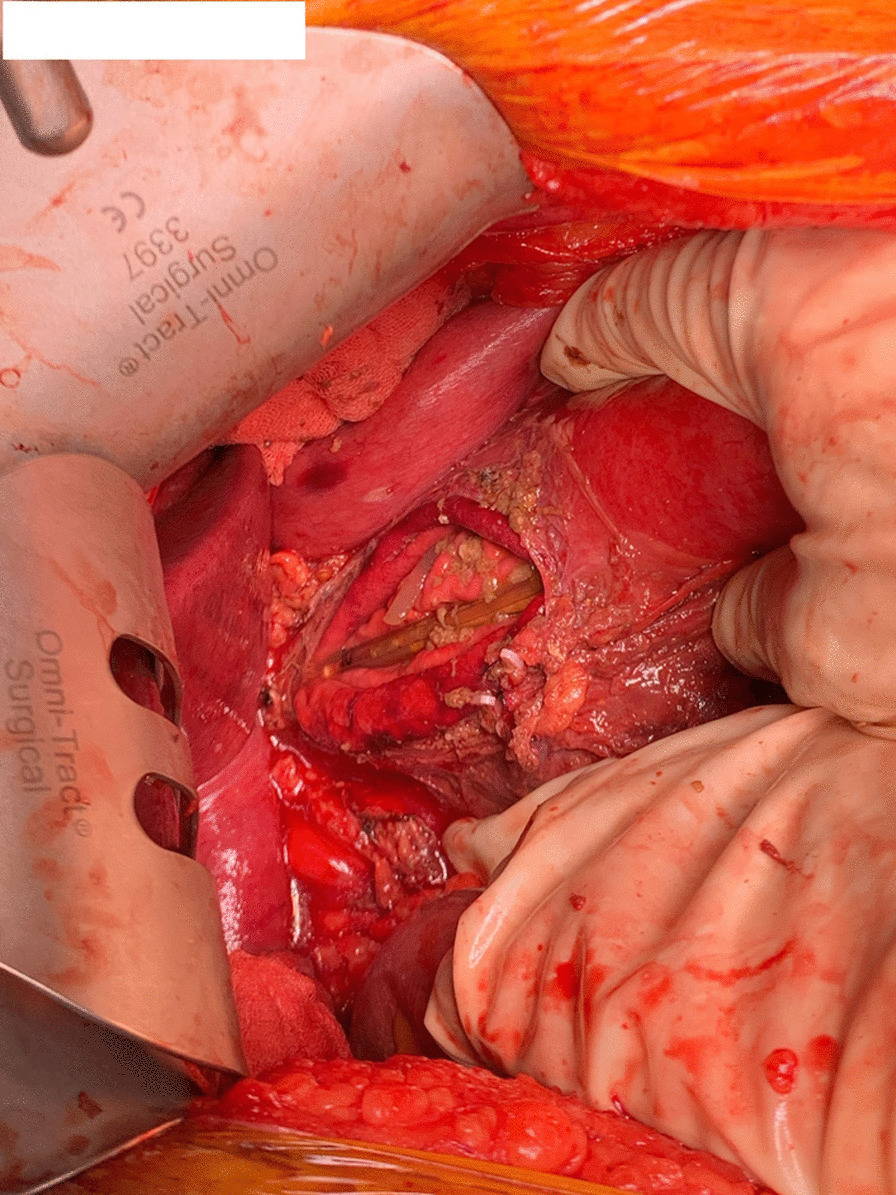
Intra-operative photo showing linear tear along lesser curvature. The decompressive nasogastric tube can be visualised in the stomach, through the tear

A midline laparotomy revealed a significant amount of contaminated fluid in the lesser curvature around the gastro-hepatic ligament and in the abdominal cavity. The gastro-hepatic ligament was divided and a 6 cm long linear tear along the lesser curvature near the left gastric artery was found (Fig. 4). Necrotic tissue surrounding the perforation was cleared. The stomach was full of food residue and red wine. The tear was primarily repaired in two layers with absorbable (3.0 PDS) sutures and a Jackson Pratt drain was placed in the gastro-hepatic area after a washout. There was minimal blood loss. The patient was observed in the ward post-operatively. His post-operative period was prolonged due to development of a left basal pneumonia, a deep abdominal wall collection at the laparotomy site, and left back pain secondary to small splenic infarcts. All these complications were managed conservatively. He had a further uneventful course of recovery and was followed up at our clinic in 2 months after discharge.

## Methods

The literature search and review was conducted by Y.H.

The initial search was implemented on September 22, 2021, on three electronic databases: Medline (1946—present), Embase (1947—present), and PubMed (1946—present). The only limit placed was for ‘Human studies’; there were no further limits on date, language, or age selected. The search query consisted of terms such as: ‘stomach rupture’, ‘gastric rupture’, ‘binge eating’, and sodium bicarbonate’. Two search strings were formulated and ran ‘(binge eating) AND [(stomach rupture) OR (gastric rupture)] and (sodium bicarbonate) AND [(stomach rupture) OR (gastric rupture)]’. The articles were screened for cases reporting gastric/stomach rupture following ingestion of a large meal, with or without ingestion of sodium bicarbonate. The reference lists of selected articles were then manually searched to identify any other cases not yet captured. A ‘snowball’ technique was adopted in which citation within articles were search if they appeared relevant to the review.

Data including year of publication, demographic profile of the case, history of eating disorder if reported, use of sodium bicarbonate, site and size of gastric perforation, as well as the mortality outcome of the case were compiled in a single spreadsheet. Descriptive statistics were calculated to summarise the data. Nominal data is described with percentages. Table 1 summarises the cases included in our analysis.

**Table 1:** Summary of cases of spontaneous gastric rupture associated with binge-eating

No	Author	Years	Sex	Age	History of eating disorder	Sodium bicarbonate	Site of perforation	Size of perforation	Outcome
1	Murdfield [22]	1926	M	39	ND	Yes	LC	5.3 cm	Died
2	Lemmon and Paschal [20]	1941	F	51	ND	Yes	LC	12.5 cm	Died
3	Bruno et al. [23]	1963	F	58	ND	Yes	LC	6 cm	Died
4	Schwartz and Zimetbaum [24]	1966	F	65	ND	Effervescent antacid tablets	LC	4 cm	Died
5	Evans [7]	1968	F	20	AN	ND	GC (MP)	3.5 cm	Recovery
6	Matikainen [25]	1979	F	18	AN, binge/purge	ND	AW	2 cm	Died
7	Saul et al. [9]	1981	F	22	AN, binge	ND	AW	1 cm	Died
8	Mastrangelo and Moore [26]	1984	M	31	ND	Yes	LC	5 cm	Recovery
9	Edwards [27]	1985	F	23	BN	ND	SW	ND	Died
10	Lazebnik et al. [28]	1986	M	38	ND	Yes	LC	10 cm	Recovery
11	Breslow et al. [29]	1986	F	32	BN	Yes	LC	6–8 cm	Recovery
12	Abdu et al. [10]	1987	F	17	BN	ND	ND	ND	Recovery
13	Downs and Stonebridge [30]	1989	M	70	ND	Yes	LC	6 cm	Recovery
14	Beiles et al. [11]	1992	F	24	BN	ND	AW	7 cm	Recovery
15	Roseborough and Felix [31]	1994	F	43	BN	ND	LC	2 cm	Died
16	Willeke et al. [32]	1996	F	19	AN, binge	ND	PW	ND	Recovery
17	Nakao et al. [33]	2000	F	17	AN, binge/purge	ND	AW	8 cm	Recovery
18	Qin et al. [34]	2000	F	4	ND	ND	GC	7.5 cm	Recovery
19	Ishikawa et al. [35]	2003	M	49	ND	ND	AW (MP)	14 cm & 6 cm	Died
20	Turan et al. [36]	2003	M	18	Mentally retardation	ND	AW	9 cm	Died
21	Sinicina et al. [37]	2005	F	19	AN	ND	AW	15 cm	Died
22	Libeer et al. [38]	2007	F	3	ND	ND	AW	7.5 cm	Recovery
23	Morse and Safdar [39]	2007	F	18	EDNOS	ND	LC	4 cm	Recovery
24	Hattori et al. [40]	2008	F	22	BED	ND	GC	ND	Died
25	Trindade et al. [41]	2008	F	13	ND	ND	AW	5 cm	Recovery
26	Hiraga et al. [42]	2012	M	79	Cognitive impairment	ND	AW	10 cm	Recovery
27	Mishima et al. [43]	2012	M	12	ND	ND	AW	3 cm	Recovery
28	Jung et al. [44]	2012	F	23	EDNOS	ND	Fundus	ND	Recovery
29	Sahoo et al. [45]	2013	M	36	ND	ND	LC	ND	Recovery
30	Tatsuo et al. [46]	2013	F	26	AN	ND	PW (MP)	13 cm and 3 cm	Recovery
31	Dewangan et al. [8]	2016	M	17	ND	ND	AW	ND	Recovery
32	Dincel and Goksu [47]	2016	F	24	ND	ND	Antrum	ND	Died
33	Vasquez et al. [48]	2017	F	54	ND	Yes	LC	5 cm	Recovery
34	di Luca et al. [49]	2018	F	18	AN	ND	Fundus (MP)	ND	Died
35	Han et al. (present case)	2021	F	21	BED	Yes	PW	15 cm	Died
36	Han et al. (present case)	2021	M	63	ND	Yes	LC	6 cm	Recovery

*M* Male, *F* Female, *ND* Not Described, *AN* Anorexia Nervosa, *BN* Bulimia Nervosa, *EDNOS* Eating disorder not otherwise specified, *BED* Binge-eating disorder, *LC* Lesser Curvature, *GC* Greater Curvature, *MP* Multiple perforations, *AW* Anterior Wall, *SW* Stomach wall, *PW* Posterior wall

## Results

### Demographics

Gastric rupture is a rare clinical condition. In the past 100 years, only 36 cases (including the two present cases) have been reported in literature for spontaneous gastric rupture associated with a binge-eating episode. About half of the cases (47.2%) included have a current diagnosed or undiagnosed eating disorder, with anorexia nervosa being the most common (47.0%).

Gastric rupture is more prevalent in females (69%). Majority of the females included in our analysis have a history of eating disorder (68%), while none of the male patients have a history of eating disorder.

The age range of patients included in this analysis is wide, with the youngest being 3-years-old and the oldest being 79-years-old. The mean age of 31-years-old. Gastric rupture is associated with high mortality (41.6%).

There are 10 cases reported in literature (including the two present cases) of gastric rupture associated with sodium bicarbonate ingestion since 1926. Apart from the case reported by Vasquez et al. [48] in 2017, the last reported case was in 1989 [30].

### Site of rupture

**Fig. 5:**
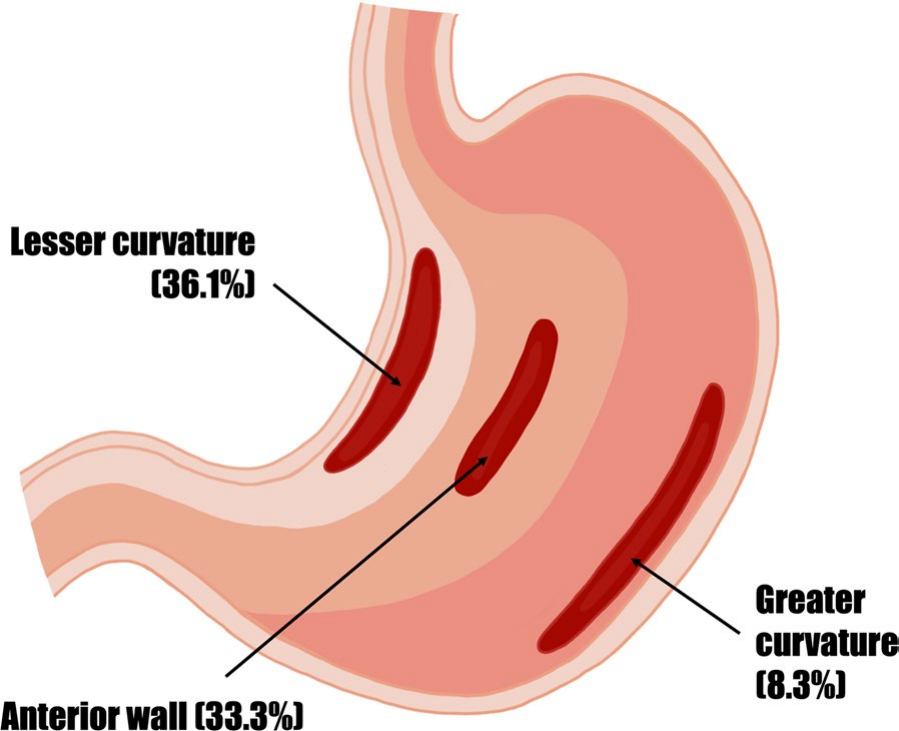
Common sites of spontaneous rupture

The most common site of gastric rupture is the lesser curvature (36.1%), and the anterior wall is the second most common site (33.3%). Posterior wall and greater curvature ruptures are uncommon, with three cases reported for each site. The sites of ruptures are illustrated in Fig. 5. The lesser curvature is the weakest part of the muscular stomach wall due to the decreased number of mucosal folds [20, 24, 30]. As the stomach become distended, it assumes a spherical shape which stretches out the lesser curvature [24, 48], causing local ischaemic necrosis and predisposing it to perforation [24]. The insertion of the gastro-hepatic ligament also results in relatively decreased mobility of the lesser curvature during distension [21, 30], contributing to its propensity to rupture. On the other hand, greater curvature ruptures are due to lacerations resulting from emesis, as compared to lesser curvature ruptures which are due to distension [25, 26, 29]. In majority of the cases, rupture occurs at a single site (88.9%). Increased intragastric pressure can occasionally cause ruptures at multiple sites [35], and in our analysis, four cases report multiple perforations. The average size of rupture was 6.9 cm.

### Associated causes

Gastric rupture is associated with some degree of gastric dilatation [25]. Associated causative factors include binge-eating episodes [24], gas release from sodium bicarbonate consumption [20, 22] or fermentation of gastric contents, proximal and distal outlet obstructions such as superior mesenteric artery syndrome, ligament of Treitz syndrome, and muscular atony [39]. Other aetiologies such as anorexia nervosa and bulimia, electrolyte disturbances, diabetes mellitus, as well as anaesthesia and debilitation have been implicated as well [44].

## Discussion

### Demographics

We found that gastric rupture is more prevalent in females, as similarly reported in previous analyses (69% vs 63–83%) [9, 33, 36]. The age range included in our analysis is wider than previously reported (3–79 years vs 14–48 years) [33]. This could be due to our wide inclusion criteria, where paediatric cases were not excluded in our analysis. The mean age of patients is also older than previously reported (31 years vs 22.9 years) [33].

### Pathogenesis

The stomach is well protected from spontaneous rupture due to its anatomy. The presence of two opening points for the venting of pressure [24] and elastic walls indicates that the stomach wall has great propensity for dilatation [20]. Hence, at least two factors are necessary for the occurrence of spontaneous gastric rupture [21]. First, there must be a sudden distension of the stomach, which produces a fixed shape, reduces mobility, and predisposes the thinned wall to rupture [21]. Second, a disturbance in the emptying mechanism of the stomach which prevents venting of the high gastric pressure [21, 24].

DEBs can lead to spontaneous rupture of the stomach. With progressive filling of the stomach, a tonic physiological relaxation first occurs [20]. Mechanical mucosal tears can occur when the stomach is filled beyond the limits of this state [20]. When dilatated, vagotomy or sympathetic stimulation causes relaxation of the gastric body and increased tension at the pyloric sphincter, resulting in decreased gastric emptying and further dilatation [42]. The stomach then becomes increasingly hypotonic and over-stretched. In this overdistended state, areas of softening and infarcts are produced as small amounts of hydrochloric acid can cause ulcers and necrosis [20]. Revilliod demonstrated in cadavers that around four litres is required for gastric rupture [30, 50]. However, bulimic patients could ingest more than 4 L, [29, 50] with the stomach harbouring more than 12 L during a binge episode reported [10, 51]. Furthermore, following a period of starvation or in anorexic patients, the stomach undergoes atony and muscular atrophy [7, 10, 52]. Scobie states that in patients with anorexia nervosa, direct neurogenic gastric paralysis secondary to malnutrition develops [53, 54]. Cell wall atrophy has also been postulated in these patients due to chronic starvation [32], and small food intake in the previous 2 years can result in gastric muscular atrophy [7]. The stress resulting from a sudden ingestion of large amounts of food can lead to gastric atony and precipitate AGD [7, 41, 52].

**Fig. 6:**
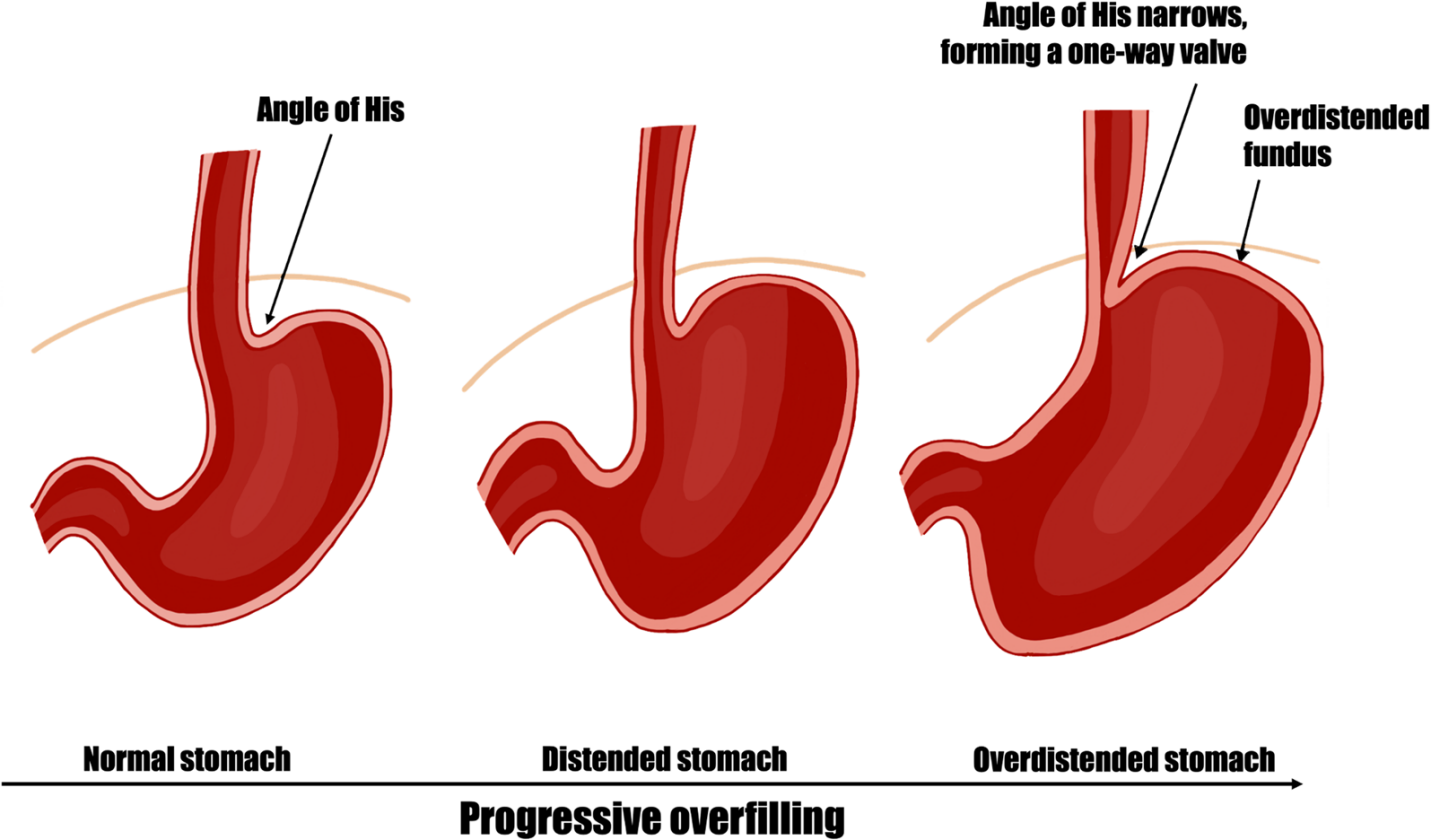
Occlusion of cardio-oesophageal junction by distended junction

Functional spasm of the pylorus or cardio-oesophageal junction can affect gastric emptying and venting of pressure [21], resulting in the development of a functional gastric outlet obstruction. Some authors suggested that AGD may be a functional entity to regional disease like pancreatitis or peptic ulcer [44, 55], while others report that AGD can occur in patients with eating disorders [44]. Overdistension of the stomach fundus may angulate the oesophageal walls against the fixed fibres of the crus of the diaphragm [56], decreasing the angle of His, and forming a one-way valve which prevents regurgitation of gastric contents into the oesophagus [24] as illustrated in Fig. 6. This results in the patient’s inability to vomit to decompress the stomach, a characteristic symptom of AGD [29]. Superior mesentery artery syndrome, which is the compression and occlusion of the horizontal duodenum between the superior mesenteric artery and the abdominal aorta [42], has been suggested as a contributing factor in emaciated patients [11, 57]. This results in distal mechanical obstruction and decreased gastric emptying. The retention of food in dilatation also stimulates more gastric secretion [38], further perpetuating the problem. The occlusion of the cardio-oesophageal junction and the distal gastric outlet in an already distended stomach predisposes the stomach to rupture with any sudden increase in intragastric pressure [24]. This can be from coughing, attempting to vomit, or gas release from fermented food or the reaction of sodium bicarbonate with hydrochloric acid [24, 58].

There is debate surrounding the role of sodium bicarbonate in spontaneous gastric rupture. Fordtrand et al. [59] concluded that gas production from the reaction of ingested sodium bicarbonate and gastric acid is slow, and recommended that the dose of sodium bicarbonate for relief of dyspepsia should be half a teaspoon. However, most people exceed this recommended dose [28]. Murdfield et al. experimented with cadavers and showed that sudden gas release from the addition of sodium bicarbonate to 2–3 L of dilute hydrochloric acid will regularly produce rupture of stomach [20, 22].

Gastric dilatation can cause gastric necrosis, and ischaemia generally occurs before rupture [36]. Several theories have been proposed. According to Wolloch and Dinstman, direct mucosal necrosis occurs with gastric dilatation [10, 60]. Venous congestion is described as a cause, when the sudden increase in intragastric pressure beyond 20 cm of H_2_O [61, 62] exceeds gastric venous pressure [9, 61], decreasing intramural venous flow, and results in ischaemia and infarction [44]. There have not been any documentation of gastric infarction due to arterial insufficiency, due to the extensive collateral circulation of the stomach [9, 31, 63]. It has been said that occlusion of the four major arterial trunks and 80% of the smaller collateral arteries supplying the stomach is not sufficient to cause infarct [9, 41, 61].

### Mortality

While the mortality of gastric rupture has improved compared to previously reported mortality rates (41.6% vs 73–100%) [9, 36, 45], the high mortality rate is still significant. In comparison, the 60-days in-hospital mortality after surgery for a perforated ulcer in the United Kingdom between 2013 and 2015 was 11.7% [64]. Causes of death from spontaneous gastric rupture could be due to shock (hypovolaemic, neurogenic, or septic) [27, 36, 37], aspiration [27], arterial oxygenation insufficiency [27, 49], or disseminated intravascular coagulopathy [31]. Acute abdominal distension inhibits venous return via the inferior vena cava [27], resulting in profound hypotension and reduced cardiac output, with reversal after gastric decompression described [65]. Splanchnic vessel sequestration and splanchnic nerve mediation has been proposed as a contributory pathogenesis as well [36]. Neurogenic shock caused by extreme gastric dilatation has been described, which is a distributive shock due to paradoxical interruption of sympathetic excitation associated with parasympathetic excitation [37]. Contamination of the peritoneal cavity can lead to profound sepsis [66]. In bulimic patients conditioned to self-induced vomiting, a lack of oesophageal sphincter tone [27] and high intragastric pressure in a distended stomach increases the risk of an aspiration event. Massive abdominal distension leading to upward displacement of the diaphragm causes compression of the heart and reduction of lung vital capacity, which can consequently cause heart and respiratory failure [49]. Disseminated intravascular coagulopathy has been described in several patients with AGD and rupture [31], including our first case. A large amount of tissues could be ischaemic until gastric decompression, secondary to hypotension or reduced perfusion pressure of the abdominal or the lower limbs. Disseminated intravascular coagulopathy secondary to reperfusion injury can occur [67], leading to irreversible and uncontrollable haemorrhage and death.

### Management

AGD without perforation can be managed conservatively in some cases [45, 47]. However, with gastric perforation, definitive surgical management is necessary [36, 45, 47].

Despite the rarity of the clinical condition, a high degree of suspicion should be maintained in patients presenting with severe abdominal pain after ingestion of a heavy meal. This is especially so in patients with a history of eating disorders, use of sodium bicarbonate for relieve of dyspepsia, or recent starvation.

Imaging studies such as abdominal or chest X-ray may reveal gas distension and subdiaphragmatic free air in cases with perforation [45, 47]. Abdominal CT remains the most useful imaging modality for diagnosis and revealing of aetiology [10, 47]. Standard resuscitation protocols with intravenous fluid resuscitation [47], early antibiotic therapy in septic patients [66], as well as attempting gastric decompression can improve outcomes. This can be done with a nasogastric tube, gastric needle decompression as described in this case, or upper gastrointestinal endoscopy [47].

Ultimately, early operation is required [47] to decompress the stomach [45], excise necrotic tissue, and lavage peritoneal cavity to reduce chemical peritonitis and sepsis [66]. In patients at risk of ischaemic injury to the bowel, a ‘second-look’ laparotomy at 24–48 h has long been practiced in ischaemic small bowel conditions and may be of value [11].

### Relevance

This paper adds to the current limited literature surrounding spontaneous gastric rupture associated with binge-eating. Less than 50 cases have been reported in the past 100 years, and this is only the second and third case to be reported in Australia. Spontaneous gastric rupture, while rare, can affect people anywhere in the world. In our analysis, studies are reported across the globe, in all populated continents except for Africa. This could be due to the lack of reporting or lack of recognition of the condition.

Moreover, some patients, particularly those with bulimia nervosa, appeared to be more vulnerable to the impact of lockdown secondary to COVID-19, resulting in increased pathological eating behaviour including binge-eating [68]. Pervasive media coverage about threats of food shortages [69] and intense use of social media during lockdown are contributory factors to relapses in DEB [70], and the stress and anxiety associated with the pandemic also play a part [71].

The combination of binge-eating and the uneducated use of home remedies could prove to be dangerous and potentially fatal, as described by the cases we report. More research is required in this area for recommendations in clinical practice.

## Conclusion

Due to the potentially fatal consequence of gastric rupture, there should be a low threshold of suspicion for a patient presenting with severe abdominal pain with abdominal distension following an episode of binge-eating. This is especially if they have a history of eating disorder, period of starvation, or ingested gas-producing antacids such as sodium bicarbonate. There is a need for patient education around the use of OTC medications or home remedies, as patients are often unaware of the potentially fatal adverse effects associated with inappropriate use.

## Data Availability

Not applicable.

## Abbreviations

DEB: Disordered eating behaviour
AGD: Acute gastric dilatation
OTC: Over-the-counter
CT: Computed tomography
